# Practical Gynecology, Obstetrics and the Menopause

**Published:** 1903-04

**Authors:** 


### BOOK REVIEWS.

Practical Gynecology, Obstetrics and the Menopause. By A. H. P. Leuf, M. D., Philadelphia. Three Parts, complete in one volume of 326 pages. Published by the Medical Council, Philadelphia, Pa. 1902. [Price, $2.50.]

When the writer of this, which for courtesy's sake is called a review, was handed Dr. Leuf's maiden work on "Practical First Principles" he announced it as a literary curiosity and, in spite of the fellow-feeling which is supposed to exist among associate editors, was constrained to say briefly that much good book paper had been wasted, that the book was hopelessly useless. It was in nowise intended to discourage Dr. Leuf's literary efforts; nor was it expected that he would discontinue his reckless sowing of literary wild oats. Yet in spite of the gentle admonition he has sent forth into an unfeeling and unsympathetic world another volume,—a sort of trinity, titled as above and divided into three parts, which is stated to be "a revised and enlarged reissue of three serial articles appearing in The Medical Council."

The fact that the book is published by The Medical Council makes it easy for Dr. Leuf, for thereby he divides the responsibility for this, his newest bit of careless wastefulness of good paper and binding.

Somehow or other we should miss the bookishness of a collection of printed pages bound in board covers if there were no preface, and Dr. Leuf very graciously gives us a preface which serves a triple purpose,—this being a triple sort of book. It makes the volume complete from a mechanical standpoint; it apologises for its appearance, and it serves to soften the shock of the table of contents which follows. Then, so that we may not miss anything and at the same time be properly impressed with the utter bookishness of the volume as well as the debonair literary atmosphere of the author, we are treated to a Foreword and likewise an Introduction, and then we are plunged into the chapters of Part I.—The General Practitioner as his own Gynecologist, which in itself is an anatomic curiosity when one considers it impassionately.

In his preface, which would be actually criminal to pass over, Dr. Leuf lays bare his heart and says his book is "just as deficient as my own experience has been." A coarse and unfeeling reviewer with little of the milk of human kindness would stop right here, lay down his pen and say something unkind; but the book has a purpose,—it may be to serve as a warning to too enthusiastic young men with literary pruritis,—and we shall let the author tell about it. He is frank about the purpose too, for in the gynecologic section he admits his aim has been to encourage the general practitioner not to send his cases to the specialist. In the Foreword,—we must not pass over the Foreword either,—he says: "Gynecology is easy of practice; more so than many of the other branches of medicine," and then in graceful strides he tears into his introduction and proceeds to lay bare the secrets which have been imprisoned in his soul's soul. He abhors specialists,—inferentially they are humbugs,—and he says it in language which has the twang of the man who advertises to cure all ills by the laying on of hands. He strays far from the rosy paths of literature where he may gather fragrant blooms, to wallow in the marsh and wander around in an endeavor to bespatter the "specialist." It would be giving him undue and undeserved prominence besides being a waste of valuable space to quote his shrill warnings, yet it would be just punishment, for they read like a patent medicine circular promising a ready road to a rapid and ruddy restoration to health.

As regards the practice advocated by the author,—everyone is an author who writes a book,—little need be said. He doesn't use metallic sutures any more; he uses silkworm-gut and holds it in place with perforated shot. If one would follow his teachings he must needs have an "operating pad. . . . They bring patients to the men who have them." Generally speaking, the tone of the book is egotistic. It deserves a place beside Dr. Leuf's previous work. N.W.W.

